# Simple and Sensitive Detection of Bacterial Hydrogen Sulfide Production Using a Paper-Based Colorimetric Assay

**DOI:** 10.3390/s22155928

**Published:** 2022-08-08

**Authors:** Byung-Ki Ahn, Yong-Jin Ahn, Young-Ju Lee, Yeon-Hee Lee, Gi-Ja Lee

**Affiliations:** 1 Department of Biomedical Engineering, College of Medicine, Kyung Hee University, Seoul 02447, Korea; 2 Department of Orofacial Pain and Oral Medicine, Kyung Hee University Dental Hospital, Kyung Hee University School of Dentistry, Seoul 02447, Korea; 3 Department of Medical Engineering, Kyung Hee University Graduate School, Seoul 02447, Korea

**Keywords:** hydrogen sulfide, bacteria, paper, colorimetric assay

## Abstract

Hydrogen sulfide (H_2_S) is known to participate in bacteria-induced inflammatory response in periodontal diseases. Therefore, it is necessary to quantify H_2_S produced by oral bacteria for diagnosis and treatment of oral diseases including halitosis and periodontal disease. In this study, we introduce a paper-based colorimetric assay for detecting bacterial H_2_S utilizing silver/Nafion/polyvinylpyrrolidone membrane and a 96-well microplate. This H_2_S-sensing paper showed a good sensitivity (8.27 blue channel intensity/μM H_2_S, R^2^ = 0.9996), which was higher than that of lead acetate paper (6.05 blue channel intensity/μM H_2_S, R^2^ = 0.9959). We analyzed the difference in H_2_S concentration released from four kinds of oral bacteria (*Eikenella corrodens*, *Streptococcus sobrinus*, *Streptococcus mutans*, and *Lactobacillus casei*). Finally, the H_2_S level in *Eikenella corrodens* while varying the concentration of cysteine and treatment time was quantified. This paper-based colorimetric assay can be utilized as a simple and effective tool for in vitro screening of H_2_S-producing ability of many bacteria as well as salivary H_2_S analysis.

## 1. Introduction

Hydrogen sulfide (H_2_S) is the third member of small gaseous transmitters (or gasotransmitters) family, together with nitric oxide and carbon monoxide. The abnormal concentration of H_2_S may correlate with many diseases including Alzheimer’s disease [1], Parkinson’s disease [2], liver diseases [3], acute pancreatitis [4], and diabetes [5]. Moreover, high concentration of H_2_S in oral cavity has also been linked to the progression of oral diseases such as halitosis, gingivitis, and periodontitis [6,7,8]. In particular, the increase in H_2_S level as a result of the accumulation of pathogenic bacteria can facilitate periodontal diseases such as gingivitis and periodontitis [8], which have been known to be associated with anaerobic and proteolytic bacterial metabolism [9,10,11]. Oral malodor is mainly due to putrefactive actions of oral bacteria producing volatile sulfur compounds (VSC)—including H_2_S, methyl mercaptan, and dimethyl sulfide—on endogenous or exogenous proteins and peptides [9]. Therefore, it is necessary to quantify H_2_S produced by oral bacteria for the diagnosis and treatment of oral diseases.

Conventional methods for detection of bacterial H_2_S include gas chromatography [12] and spectrophotometric analysis based on methylene blue (MB) assay [11] or bismuth sulfide (BS) assay [13]. Although gas chromatography-based analysis was highly sensitive with a limit of detection (LOD) of 1.6 ng/mL [12], it needed special instrument and complicated sample pretreatment. In addition, MB and BS assays had a main drawback of a relatively low sensitivity. Basic et al. [11] reported that the visual detection limit for H_2_S was 0.6 mM for the BS assay and 2 mM for the MB assay. In addition, Zhu and Chu [14] suggested a simple visual method to detect H_2_S in bacteria using a modified version of BS precipitation that used 96-well plates. However, its sensitivity was low with 0.2 mM of visual detection limit. Lead acetate paper strip, which reacts with H_2_S to produce a brown lead sulfide, has been utilized to monitor H_2_S production in bacteria [15,16]. Although lead acetate paper is simple, easy to use, and cost-effective, it needs to use a toxic chemical.

Paper-based analytical devices (PADs) have received great attention for point-of-care applications including clinical analysis, food safety, and environmental assessment to improve human health [17,18,19]. According to the World Health Organization (WHO), low-cost sensors for use in developing countries must fulfill the “ASSURED” criteria which stands for Affordable, Sensitive, Specific, User-friendly, Rapid and robust, Equipment-free, and Delivered to end-users [19,20]. Among them, user-acceptance such as UED determine the commercial potential of the devices [20]. The main advantages of PADs are affordability, portability, and disposability. In addition, they can be designed with sufficient sensitivity and specificity for field use [20], thus can satisfying the “ASSURED” criteria. Our previous report has shown that a paper-based colorimetric assay utilizing silver/Nafion™/polyvinylpyrrolidone (Ag/Nafion/PVP) membranes could quantify endogenous H_2_S released from living cancer cells [21]. This paper assay, fabricated with a 96-well microplate type, possessed good sensitivity, high selectivity, and good stability, as well as excellent reproducibility. Moreover, we successfully detected H_2_S from 3D-culture, live cancer cells using a H_2_S sensing paper with 4 circles which was modified to fit the paper-integrated analytical device [22].

**Figure 1:**
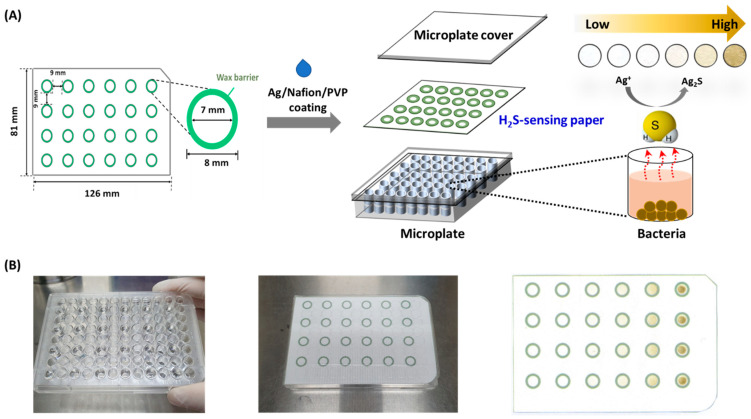
(**A**) Schematic illustration of the fabrication process of an Ag/Nafion/PVP based H_2_S-sensing paper and experimental design for colorimetric detection of bacterial H_2_S; (**B**) Photographic images of the paper-based colorimetric assay and the H_2_S-sensing paper after reaction with H_2_S.

In this study, we analyzed bacterial H_2_S production from four kinds of oral bacteria including *Eikenella corrodens* (*E. corrodens*), *Streptococcus sobrinus* (*S. sobrinus*), *Streptococcus mutans* (*S. mutans*), and *Lactobacillus casei* (*L. casei*) that could be cultured under aerobic condition utilizing an Ag/Nafion/PVP-coated paper. This paper was patterned with 24 multi-zones which were fitted to a diameter of each well in a microplate and spaced apart at regular intervals. The color of Ag/Nafion/PVP on the paper was changed by free H_2_S gas, because it reacted with Ag ion to form a brownish Ag_2_S. First, we re-optimized compositions of coating solutions to maximize the color change in the detection zone on the wax-patterned paper. We then compared the analytical performance of this H_2_S-sensing paper with that of lead acetate paper as a reference. Finally, we measured H_2_S released from four kinds of oral bacteria. The concentration of H_2_S in *E. corrodens* was quantified by varying the concentration of L-cysteine (Cys) and treatment time. The experimental scheme is presented in Figure 1A.

## 2. Materials and Methods

### 2.1. Chemicals

PVP (K90), silver nitrate (AgNO_3_, ≥99.0%), Nafion™ perfluorinated resin solution, sodium sulfide (Na_2_S), Cys (≥98.0%), L-homocysteine (H-Cys, ≥98.0%), dithiothreitol (DTT), and reduced L-glutathione (GSH) were purchased from Sigma Aldrich (St. Louis, MO, USA). Columbia broth and sheep blood defibrillated were obtained from MB cell (Seoul, Korea). Brain heart infusion (BHI) broth and De Man, Rogosa and Sharpe (MRS) broth were purchased from Difco Lab. Inc. (Detroit, MI, USA). All chemicals and reagents were of an analytical grade. They were used as received without further purification. All aqueous solutions were prepared with de-ionized water (DW) of 18.3 MΩ/cm resistivity.

### 2.2. Preparation of H_2_S-Sensing Paper

A paper substrate (width: 126 mm; length: 81 mm; thickness: 0.18 mm in thickness) based on Whatman^®^ filter paper (Grade 1; GE Healthcare Bio-Sciences, Pittsburgh, PA, USA) was designed using AutoCAD. It contained 24 circular detection areas with each of an inner diameter of 7 mm (Figure 1), which was larger circle than that in our previous report [21]. To diminish unwanted error caused by gas diffusion, sensing zones were spaced 9 mm apart. A Xerox ColorQube™ 8570N printer (Fuji Xerox, Tokyo, Japan) was used to pattern hydrophobic wax barriers. The wax-patterned paper was heated in a BF-150C drying oven (DAIHAN Scientific, Seoul, Korea) at 130 °C for 90 s for uniform impregnation of wax. Finally, the paper was pulled from the oven and cooled to room temperature (RT).

The H_2_S sensing paper was fabricated as described previously [21,23]. Briefly, PVP (5% *w*/*v*) solution was mixed with Nafion™ in the ratio of 10:0, 9:1, 6:4, 4:6, and 0:10 (*v*/*v*). Then, 30 μL of AgNO_3_ solution (0.025, 0.05, 0.1, 0.2, and 0.4 M) was added into 1 mL of Nafion™/PVP mixture, respectively, and mixed well using a vortex mixer. After 20 μL of the mixture was dropped on each detection zone in the wax-patterned paper substrate, the paper substrate was dried in a clean room (23.5 ± 1.0 °C, 25.0 ± 5.0% humidity) for at least 3 h.

### 2.3. Evaluation of Analytical Performance of the H_2_S-Sensing Paper

A standard solution of Na_2_S as H_2_S donor with different concentrations (6.25, 12.5, 25, and 50 μM) was prepared with 100 mM phosphate buffered saline (PBS, pH 7.4). The analytical performance of the H_2_S sensing paper was evaluated using previously reported procedures [21,24]. Briefly, a 300 μL of Na_2_S solution with each concentration was added into each well of a microplate which was matched on the detection zone in the paper. In addition, the H_2_S-sensing paper was placed on the Na_2_S-loaded microplate and covered with a microplate lid. Figure 1B show photographic images of the paper-based colorimetric assay. H_2_S gas formed from Na_2_S was reacted with this paper for 1 h at RT. Considering the two-step dissociation of H_2_S and equilibrium coefficients (K_1_ and K_2_) [25], the actual H_2_S concentration was converted to about 0.33 times the Na_2_S concentration. After reaction with H_2_S for 1 h, the H_2_S-sensing paper was taken out from the microplate. Color changes in this paper were firstly confirmed with naked eyes and the image was subsequently obtained using an Epson scanner (Perfection V700 Photo flatbed scanner, Seiko Epson, Nagano, Japan). The blue channel intensity of the circular area (4.5 mm in diameter) on each detection zone was measured using ImageJ (National Institutes of Health, Bethesda, MD, USA) [21,22]. All values of the blue channel intensity were displayed as corrected blue channel intensity by subtracting the measured value on the detection zone from the intensity of blank zone in the Ag/Nafion/PVP-coated paper.

To compare the sensing performance of the Ag/Nafion™/PVP-coated H_2_S-sensing paper with that of a lead acetate paper as a reference, we measured the change in blue channel intensity of the lead acetate paper after reaction with H_2_S gas. The standard solution of Na_2_S (300 μL) with the same concentrations ranging from 6.25 to 50 μM was added into each well of a 96-well microplate. The lead acetate paper was also placed on the Na_2_S-loaded 96-well microplate and covered with a lid. After exposure to H_2_S for 1 h at RT, the lead acetate paper was analyzed using the same methodology described above.

### 2.4. Bacterial Strains and Culture Conditions

Four kinds of bacteria including *E. corrodens* (KCTC15198)*, S. sobrinus* (KCTC5134)*, S. mutans* (KCTC5365), and *L. casei* (KCTC3109) were purchased from Korea Collection for Type Cultures (KCTC, Jeongeup, Jeollabuk-do, Korea). *E. corrodens* was grown in a Columbia broth supplemented with 5% sheep blood at 37 °C for 40 h. *S. sobinus* and *S. mutans* were grown in BHI broth at 37 °C in a 5% CO_2_ incubator for 18 h. *L. casei* was grown in a MRS broth at 37 °C in a 5% CO_2_ incubator for 18 h. Following incubation, bacteria were centrifuged at 6000× *g* for 3 min and supernatants were discarded. *S. sobrinus*, *S. mutans*, and *L. casei* pellets were resuspended in PBS to approximately 1 × 10^8^ CFU/mL. For *E. corrodens*, bacteria were resuspended in 1 mL of DW to lyse blood and re-centrifuged at 3000× *g* for 3 min twice. The supernatant was discarded and *E. corrodens* pellet was resuspended in sterilized saline to approximately 1 × 10^8^ CFU/mL.

### 2.5. Detection of Bacterial H_2_S Using the H_2_S-Sensing Paper

Prepared bacterial suspensions (1 × 10^8^ CFU/mL) of four kinds of bacteria were serially diluted to 1 × 10^1^, 1 × 10^2^, 1 × 10^4^, and 1 × 10^6^ CFU/mL. A 300 μL of each bacterial suspension was transferred to a 96-well microplate. The plate was covered with the H_2_S-sensiing paper and the lid. After incubation at 37 °C for 6 and 24 h, respectively, the H_2_S-sensing paper was analyzed using the methodology described above. In addition, 20 mM Cys was added into each bacterial suspension of four kinds of bacteria (1 × 10^8^ CFU/mL). A 300 μL of each bacterial suspension with Cys was transferred to a 96-well microplate. After incubation at 37 °C for 1 h, the H_2_S-sensing paper was analyzed using the same method.

Next, to investigate the effect of Cys on bacterial H_2_S production in *E. corrodens*, bacterial suspensions (1 × 10^8^ CFU/mL) were treated with various concentrations of Cys (0, 5, 10, and 20 mM). Then 300 μL of each bacterial suspension with Cys was loaded into each well and reacted for 1.5 h. After 10 mM Cys was added to *E. corrodens*, they were incubated for 0.5, 1, 1.5, and 2 h, respectively. Color changes in H_2_S-sensing paper were measured using a scanner and ImageJ.

## 3. Results

### 3.1. Fabrication of H_2_S-Sensing Paper

**Figure 2:**
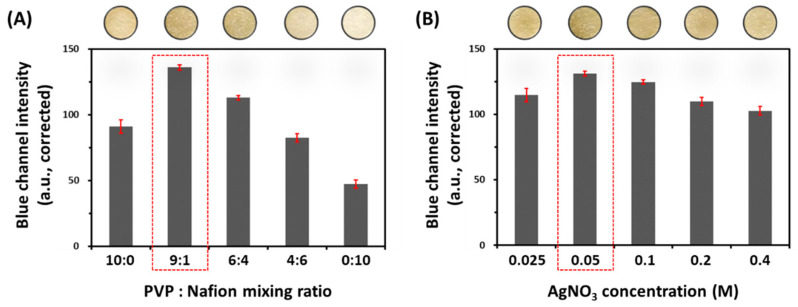
Changes in color intensity of the H_2_S sensing paper after exposure to Na_2_S (50 μM) according to (**A**) the mixing ratio of PVP and Nafion; (**B**) the concentration of AgNO_3_ concentration in the coating solution.

PADs provide several advantages, including low manufacturing cost, simplicity, portability, small sample volume, and ease of handling, making them attractive for use in many applications including clinical diagnostics and food safety analysis [17]. In addition, a white paper is a good substrate for colorimetric detection because it gives strong contrast with a color, allowing results to be assessed directly by naked eyes [26]. To apply paper as an analytical tool for the detection of bacterial H_2_S, we designed a multi-zone patterned form that was easily fitted to each well in the microplate. Next, we introduced an Ag/Nafion/PVP membrane to 24 detection zones on the paper. Although we optimized the mixing ratio of PVP and Nafion, as well as the concentration of AgNO_3_ in our previous report [21], we re-optimized compositions of the Ag/Nafion/PVP solution in the paper substrate because the diameter of detection zone changed from 4 to 7 mm. First, we measured the blue channel intensity of the H_2_S-sensing paper according to the mixing ratio of PVP/Nafion at a fixed concentration (0.05 M) of AgNO_3_. As shown in Figure 2A, the mixing ratio of PVP and Nafion markedly affected the blue channel intensity of the H_2_S-sensing paper. In particular, a 9:1 ratio of PVP and Nafion showed the greatest color change in the paper after reaction with 50 µM Na_2_S. As previously reported [21], this result might be due to the fact that a small volume of Nafion in the mixture could disperse the Ag ion homogeneously and give an excellent colorimetric response. However, the more Nafion in the coating solution, the better the invasion into the hydrophobic wall, subsequently causing a weak color change. Therefore, we selected a 9:1 ratio of PVP and Nafion for the H_2_S-sensing paper. Next, we optimized the concentration of AgNO_3_ to have the maximum color change in the H_2_S-sensing paper. Figure 2B indicates the change in the blue channel intensity of coating membrane according to the concentration of AgNO_3_. Based on the maximum color change in the detection zone, we selected the optimal concentration of AgNO_3_ in the PVP/Nafion (9:1) coating solution as 0.05 M. In addition, it was concordant with that in our previous report [21].

### 3.2. Analytical Performance of H_2_S-Sensing Paper

**Figure 3:**
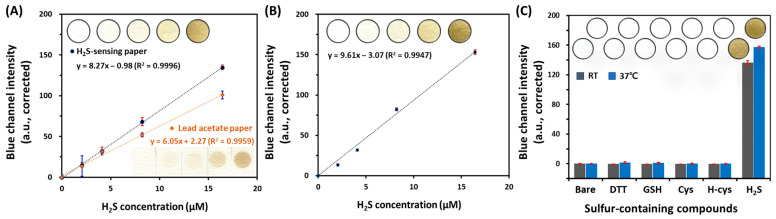
(**A**) Calibration curve of change in blue channel intensity of Ag/Nafion/PVP based H_2_S-sensing papers and lead acetate papers versus concentration of H_2_S at room temperature (RT, *n* = 4, respectively); (**B**) Calibration plot of Ag/Nafion/PVP based H_2_S-sensing papers at 37 °C (*n* = 4); (**C**) Color change in Ag/Nafion/PVP coated paper after reaction with H_2_S from Na_2_S (50 μM) and other sulfur-containing compounds (10 mM) such as dithiothreitol (DTT), reduced L-glutathione (GSH), L-cysteine (Cys), and L-homocysteine (H-Cys) at RT and 37 °C.

We evaluated the analytical performance of our H_2_S-sensing paper. Color changes in coating membranes in detection zones on the paper were analyzed after reaction with various concentrations of Na_2_S solution to each well in the microplate for 1 h at RT. As shown in Figure 3A, the calibration curve showed a good linearity over the concentration range of 2.05 to 16.4 μM for H_2_S (slope: 8.27 blue channel intensity/μM H_2_S, R^2^ = 0.9996). The LOD was 0.96 μM H_2_S (*n* = 4), based on the standard deviation of the blank (s_bl_) and the slope of the calibration curve (3 s_bl_/slope) [27]. In addition, the limit of quantification (LOQ) was found to be 3.20 μM H_2_S (*n* = 4), based on 10 s_bl_/slope.

To evaluate the feasibility of the H_2_S-sensing paper, we compared the analytical performance of our paper assay with that of the lead acetate paper as a reference. Lead acetate test paper has been utilized to detect H_2_S gas produced by microorganisms and for evaluating the quality of water and food [15,28]. Lead acetate forms a brown lead sulfide after reaction with H_2_S gas, resulting in a color change to brown. As shown in Figure 3A, the change in blue channel intensity of lead acetate paper also showed a linear relationship with H_2_S concentration ranging from 2.05 to 16.4 μM (R^2^ = 0.9959). However, its sensitivity (6.05 blue color intensity/μM H_2_S) was lower than that of our H_2_S-sensing paper (8.27 blue channel intensity/μM Na_2_S) in the same concentration range of H_2_S. The LOD and LOQ of the lead acetate paper were found to be 1.31 and 4.25 μM H_2_S (*n* = 4), respectively, which were 1.4 times higher than those of our H_2_S-sensing paper.

To examine the sensitivity of our H_2_S-sensing paper at 37 °C, we analyzed the change in blue color intensity after adding Na_2_S in the concentration range from 6.25 to 50 μM. As shown in Figure 3B, the sensitivity of this paper at 37 °C (slope: 9.61 blue channel intensity/μM H_2_S, R^2^ = 0.9947) was higher than that at RT. The LOD and LOQ were 0.23 μM (*n* = 4) and 0.70 μM H_2_S (*n* = 4), respectively. This difference in sensitivity might be attributed to the faster gas diffusion at 37 °C. Therefore, we used the calibration plot of the H_2_S-sensing paper at 37 °C to quantify the H_2_S level from bacteria.

To examine the specificity of H_2_S-sensing paper, we compared the change in blue channel intensity of this paper by Na_2_S (50 μM) with that by biologically-relevant sulfur-containing molecules (10 mM) including DTT, GSH, Cys, and H-Cys at RT and 37 °C. As shown in Figure 3C, only H_2_S gas from Na_2_S caused a great change in blue channel intensity. Therefore, this H_2_S-sensing paper detects only H_2_S gas and is not affected by other biological sulfur-containing molecules in the solution.

We also investigated the reproducibility of this H_2_S-sensing paper by evaluating changes in blue channel intensity to H_2_S in papers (*n* = 8) fabricated at different time points. As a result, the relative standard deviation was 2.86%, showing that H_2_S sensing papers were highly reproducible.

### 3.3. Detection of Bacterial H_2_S Production Using H_2_S-Sensing Paper

As oral bacteria can be biomarkers that distinguish healthy from pathological conditions within the oral cavity, oral microbiota research has potential application to develop a diagnostic and prognostic tool for human health [29]. In particular, Cys activity of oral bacteria which release H_2_S by Cys substrate can be used to check an individual’s tendency to produce oral malodor [30]. The most active oral bacteria that produce H_2_S from Cys are *Peptostreptococcus* spp., *Eubacterium* spp., *Selenomonas* spp., *Centipeda* spp., *Bacteroides* spp., and *Fusobacterium* spp. [31]. Basic et al. [11] have reported that *Fusobacterium* spp. have the most rapid and the highest production of H_2_S using both colorimetric methods such as BS and MB assays. However, some bacteria strains including *E. corrodens* and *Tannerella forsythia* known to produce H_2_S did not induce color changes with these two methods. This discrepancy might be attributed to the low sensitivity of the two colorimetric methods.

**Figure 4:**
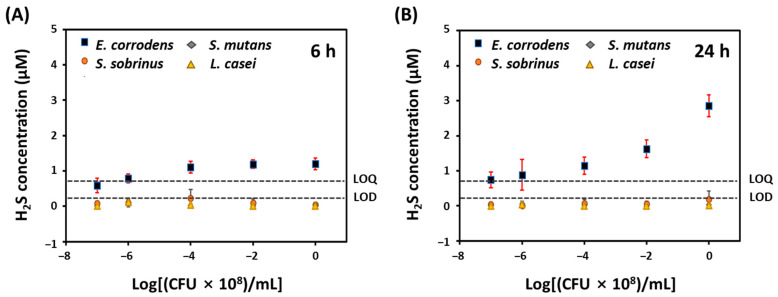
Quantitative analysis of H_2_S production from four kinds of oral bacteria such as *E. corrodens, S. sobrinus, S. mutans,* and *L. casei* according to the number of bacteria (1 × 10^1^, 1 × 10^2^, 1 × 10^4^, 1 × 10^6^, and 1 × 10^8^ CFU/mL) (**A**) for 6 h; (**B**) 24 h incubation at 37 °C.

To evaluate the effectiveness of this H_2_S-sensing paper, we measured endogenous H_2_S levels in four kinds of bacteria (*E. corrodens*, *S. sobrinus*, *S. mutans*, and *L. casei*) without any treatment. Figure 4A,B show concentrations of endogenous H_2_S released from four kinds of bacteria according to the number of bacteria (1 × 10^1^, 1 × 10^2^, 1 × 10^4^, 1 × 10^6^, and 1 × 10^8^ CFU/mL) after 6 and 24 h incubation, respectively, at 37 °C. *E. corrodens* produced a little more H_2_S than *S. sobrinus, S. mutans*, and *L. casei* after 6 h incubation. However, the H_2_S concentration produced by *E. corrodens* was similar even bacteria number increased from 1 × 10^4^ to 1 × 10^8^ CFU/mL (Figure 4A). However, as shown in Figure 4B, the change in blue channel intensity of H_2_S-sensing paper by *E. corrodens* after 24 h of incubation increased as bacteria number increased from 1 × 10^2^ to 1 × 10^8^ CFU/mL. As a result, the H_2_S concentration in 1 × 10^8^ CFU/mL of *E. corrodens* was 2.86 ± 0.31 μM based on the slope of calibration plot at 37 °C. On contrary, *S. sobrinus, S. mutan*, and *L. casei* did not produce H_2_S or they released very low levels of H_2_S below the LOD even after 24 h of incubation.

**Figure 5:**
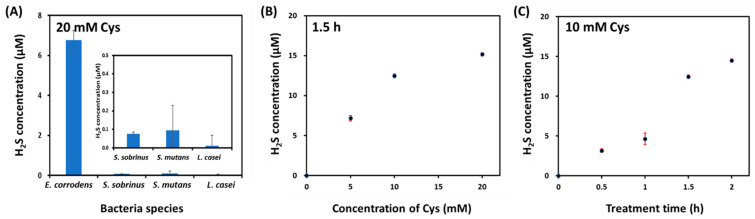
(**A**) Quantitative analysis of H_2_S production from four kinds of oral bacteria such as *E. corrodens, S. sobrinus, S. mutans*, and *L. casei* (1 × 10^8^ CFU/mL) after treatment with 20 mM Cys for 1 h. Effects of (**B**) Cys concentration (0, 5, 10, and 10 mM), and (**C**) treatment time (0, 0.5, 1, 1.5, and 2 h) on H_2_S production from *E. corrodens* (1 × 10^8^ CFU/mL) at 37 °C.

Next, to confirm the difference in H_2_S production between bacteria, we measured H_2_S concentrations in four kinds of bacteria (1 × 10^8^ CFU/mL) after treatment with 20 mM Cys as a substrate for H_2_S production for 1 h. Cys challenge test is commonly used to check H_2_S production capacity of the mouth. It is based on the fact that oral bacteria act on Cys substrate and release H_2_S in the oral cavity [30]. Cys-induced H_2_S concentration largely depends on the Cys activity of oral microbiota as well as Cys concentration [32,33]. As shown in Figure 5A, *E. corrodens* produced more H_2_S (6.76 ± 0.48 μM) after treatment with 20 mM Cys. However, *S. sobinus, S. mutans*, and *L. casei* did not produce H_2_S even after treatment with Cys. As a result, only *E. corrodens* produced H_2_S without or with treatment with Cys. To quantify the H_2_S produced in *E. corrodens* after incubation with Cys, we measured H_2_S levels in *E. corrodens* (1 × 10^8^ CFU/mL) according to the concentration of Cys (0, 5, 10, and 20 mM) and treatment time (0.5, 1, 1.5, and 2 h), respectively. As shown in Figure 5B, the production of H_2_S in *E. corrodens* increased as the Cys concentration increased at a constant treatment time of 1.5 h (7.16 ± 0.34 μM at 5 mM Cys, 12.46 ± 0.23 μM at 10 mM Cys, and 15.17 ± 0.22 μM at 20 mM Cys). *E. corrodens* also produced H_2_S in a time-dependent manner when it was incubated with 10 mM Cys (3.16 ± 0.23 μM at 0.5 h, 4.65 ± 0.72 μM at 1 h, 12.46 ± 0.23 μM at 1.5 h, and 14.48 ± 0.23 μM at 2 h) (Figure 5C). In particular, H_2_S production from *E. corrodens* could be analyzed quantitatively even after treatment with 10 mM Cys for only 0.5 h without an additional complex process. *E. corrodens* is among bacteria frequently isolated from subgingival pockets of patients with severe periodontitis [34]. From our results, we can infer that *E. corrodens* might be a member of pathogens for halitosis or bad oral breath associated with periodontitis or gingivitis.

## 4. Conclusions

We introduced a simple and sensitive colorimetric detection of bacterial H_2_S using Ag/Nafion/PVP coated paper and a 96-well microplate. This H_2_S-sensing paper showed good sensitivity, selectivity, and reproducibility. In particular, its sensitivity was 1.4 times higher than that of lead acetate paper as a reference. We successfully measured the difference in H_2_S production from different kinds of bacteria. Our Ag/Nafion/PVP coated H_2_S sensing paper exhibits some distinct advantages as follows: (1) it is easy to fabricate with a desired form; (2) it can easily and rapidly detect free H_2_S gas released from bacteria without needing a complex process, expensive instrument, or an additional time for H_2_S analysis; (3) it can sensitively and selectively detect H_2_S without needing toxic reagents such as lead acetate; (4) it can achieve the simple and high-throughput detection of H_2_S from bacteria. Therefore, our Ag/Nafion/PVP coated H_2_S-seisng paper can be utilized as a simple and an effective tool for detecting H_2_S from bacteria present in the saliva or oral cavity. Moreover, it can be applied for in vitro screening of H_2_S-producing ability of many bacteria in human body. Moreover, in the clinical field, it can be used as an auxiliary diagnostic tool with objective measurement of odor in patients with subjective halitosis and for screening oral and systemic diseases related to the increase in H_2_S level. Although our paper-based assay can partially meet the user acceptance for successful commercialization, we think that it is necessary to further improve the ease of result interpretation, as well as reduce the result readout time for H_2_S released from bacteria.

## Data Availability

The data are available from the corresponding author upon reasonable request.
